# Histological Grade and Tumor Stage Are Correlated with Expression of Receptor Activator of Nuclear Factor Kappa b (Rank) in Epithelial Ovarian Cancers

**DOI:** 10.3390/ijms23031742

**Published:** 2022-02-03

**Authors:** Raul Gomez, Miguel Á. Tejada, Víctor Rodríguez-García, Octavio Burgués, Ana I. Santos-Llamas, Andrea Martínez-Massa, Antonio Marín-Montes, Juan J. Tarín, Antonio Cano

**Affiliations:** 1 Research Unit on Women’s Health-Institute of Health Research, INCLIVA, 46010 Valencia, Spain; miguetejada85@hotmail.com (M.Á.T.); anais_santos91@yahoo.es (A.I.S.-L.); juan.j.tarin@uv.es (J.J.T.); 2 Department of Pathology, University of Valencia, 46010 Valencia, Spain; 3 Department of Pediatrics and Obstetrics and Gynecology, University of Valencia, 46010 Valencia, Spain; victorcapitol@hotmail.com; 4 Department of Pathology, Hospital Clinico Universitario, 46010 Valencia, Spain; octavioburgues@gmail.com; 5 Service of Obstetrics and Gynecology, Hospital Clínico Universitario, Av Blasco Ibáñez 17, 46010 Valencia, Spain; andrea.mm913@gmail.com (A.M.-M.); antoniomarinmontes91@gmail.com (A.M.-M.); 6 Department of Cellular Biology, Functional Biology, and Physical Anthropology, University of Valencia, 46100 Burjassot, Spain

**Keywords:** epithelial ovarian cancers, RANK, immunohistochemistry, tumor stage, tumor grade

## Abstract

The receptor activator of nuclear factor kappa B (RANK) is becoming recognized as a master regulator of tumorigenesis, yet its role in gynecological cancers remains mostly unexplored. We investigated whether there is a gradation of RANK protein and mRNA expression in epithelial ovarian cancer (EOC) according to malignancy and tumor staging. Immunohistochemical expression of RANK was examined in a cohort of 135 (benign *n* = 29, borderline *n*= 23 and malignant *n* = 83) EOCs. Wild type and truncated RANK mRNA isoform quantification was performed in a cohort of 168 (benign *n* = 26, borderline *n* = 13 and malignant *n* = 129) EOCs. RANK protein and mRNA values were increased in malignant vs. benign or borderline conditions across serous, mucinous and endometrioid cancer subtypes. Additionally, a trend of increased RANK values with staging was observed for the mucinous and serous histotype. Thus, increased expression of RANK appears associated with the evolution of disease to the onset of malignancy in EOC. Moreover, in some EOC histotypes, RANK expression is additionally associated with clinicopathological markers of tumor aggressiveness, suggesting a role in further progression of tumor activity.

## 1. Introduction

Ovarian cancer is the deadliest gynecological pathology and the seventh most lethal cancer worldwide among women [1]. About 90% of all ovarian cancers are of the epithelial type, of which serous (70–80%), endometrioid (10%) and mucinous (3–6%) are the most prevalent subtypes [1]. Survival drops dramatically at later stages of cancer development, highlighting the importance of early detection. Precise characterization of tumor features [2] and improved treatment monitoring using appropriate biomarkers [3] are emerging as effective aids to improve prognosis. Nonetheless, current pharmacological treatments for ovarian cancer have reduced efficacy, driving demand for new therapeutic agents [4]. 

Receptor activator of nuclear factor kappa B (RANK) and its ligand, RANKL, form a system belonging to the tumor necrosis factor (TNF) family of cytokines [5]. Activation of the TNF pathway induces pleiotropic effects, including inflammation, organogenesis, apoptosis and immunological functions [6,7]. The human RANK gene (TNSFR11A) consists of 10 exons and its primary major transcript codes for 616 amino acid residues (wild type [wt]-RANK), a type I transmembrane receptor which activates NF-KB upon RANK-L binding. Alternative splicing generates much less intense expression of RANK isoforms, lacking exons 9 (TNFRSF11A_∆9), 8–9 (TNFRSF11A_∆8,9) and 7–9 (TNFRSF11A_∆7,8,9), which respectively code for inactive (RANK-a), active (RANK-b) and dominant-negative (RANK-c) regulators of wt-RANK-induced NF-kB activation [8]. 

Originally, RANK was described as a key regulator of bone remodeling and breast epithelial proliferation [9]. The potential of targeting this system was initially explored for the treatment of certain related hormone-dependent pathologies like osteoporosis [10,11]. In recent years, RANK has been revealed as a master regulator of tumorigenesis [12], and its therapeutic potential in cancer has made it an attractive option. In this vein, inhibitors of the RANK/RANKL system are currently being assayed in several cancer clinical trials [13,14,15], and the list is growing. Paradoxically, despite abundant data on the involvement of RANK/RANKL in cancer development and therapy, its role in gynecological benign or malignant tumors has, until recently, remained unexplored. Our study [16] and another [17] broke new ground in showing that RANK expression is a poor prognostic factor in endometrial cancer. Moreover, we recently demonstrated a gradation in RANK protein expression between normal eutopic endometrium, endometrioma and endometrioid ovarian cancer [18]. The fact that endometrioma can be viewed as a precursor of endometrioid ovarian cancer [19] underlines the value of exploring whether RANK expression might be related to the malignant profile in this histotype of epithelial ovarian cancers (EOC), considering the other two main EOC histotypes, serous and mucinous. 

This study, therefore, has two main objectives: (i) to investigate whether RANK expression shows a gradation between the three tumor categories (benign, borderline and malignant) of the EOC histotypes (serous, mucinous and endometrioid) and (ii) to explore whether the RANK expression is related to tumor stage within the respective malignant tumor categories of each histotype. For such purposes, we designed a comprehensive methodological approach using both IHC and quantitative gene expression to investigate RANK in primary tumors with available clinicopathological features. In a more specific analysis, we explored the potential association with gene expression of specific RANK isoforms.

## 2. Results

IHC conditions were initially set up using Giant Cells of the Bone Tumor (GBCT) sections. The signal was clear and well defined in positive controls and absent when the primary antibody was omitted (Supplementary Figure S1). Staining of normal ovarian sections contained in the TMA revealed that RANK was mostly located in the vessel and scarcely in stromal cells (Supplementary Figure S2). As additional control tissues, normal fallopian tubes and endometrial samples from our archives were also immunostained against RANK. The pattern of staining observed is described in subsequent sections below. In regards to RANK QF-RT-PCR expression, quantitative analysis showed the wt-RANK (TNFRSF11A) as the highest expressed isoform in all three EOCs. Detected in most of the samples (either benign or pathologic origin), analyzed at Ct values ranging from 25–32, showed 4–7-fold higher expression than truncated TNFRSF11A_Δ8,9 or TNFRSF11A_Δ7,8,9 isoforms. TNFRSF11A_Δ9 was the lowest expressed isoform and rarely detected (Ct > 40) (Supplementary Figures S3–S5). Comparison of truncated RANK mRNA isoform values amongst tumor categories (benign, borderline and malignant) was not possible, as the expression of these isoforms was below the detection limit in most (24 out of 26 undetected; 92.2%) of the benign samples. 

Representative images of immunohistochemical staining and quantitative analysis of RANK expression at the protein and mRNA levels are described below, grouped by EOC histotype.

### 2.1. Serous EOC

#### 2.1.1. Immunohistochemical Analyses

**Figure 1:**
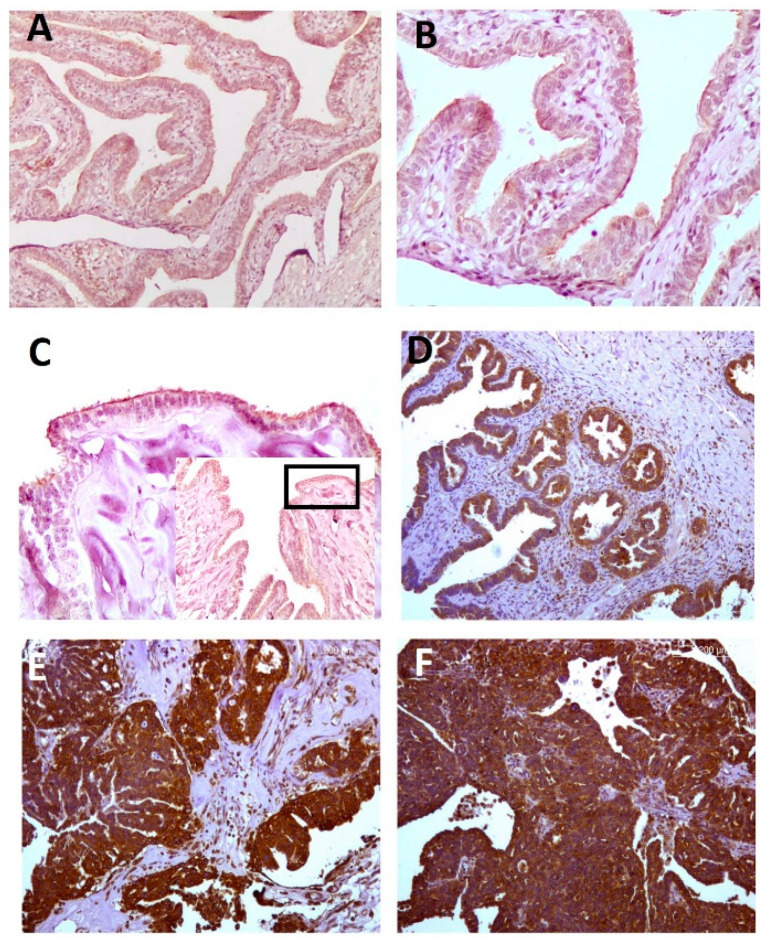
RANK staining in serous EOCs. Representative patterns of RANK immunohistochemical staining (brown) in sections classified attending to tumor category as normal (fallopian tubes: (**A**,**B**)), benign (serous cystadenoma, (**C**)), borderline (serous borderline, (**D**)) and malignant (low-grade serous carcinomas (**E**)). Additionally, malignant high-grade serous carcinomas are also shown (**F**). Note, overall signaling is mostly located in the apical region of epithelial cells in control (**B**) and benign conditions (inset detail in (**C**)) for showing moderate cytoplasmic staining. Signal intensity is brighter in malignant tissues, whereas inflammatory and mesenchymal cells of stroma present with less intense expression. Magnifications: ×100. Scale bar: 200µm.

Fallopian tubes under physiological conditions were used as a control reference to contrast RANK staining in serous EOC. In tubes, mild staining was denoted with signaling mostly restricted to the apical part of the luminal epithelium. Very few cells of the stroma presented an identifiable signal, and this was located at the perinuclear region. The staining pattern and intensity observed in tubes were mimicked in benign samples with the cytoplasmic signal present in the apical region of the epithelial cells. A notable increase in signaling was observed in borderline serous and samples, with substantially enhanced staining in undifferentiated areas of malignant serous tissues (Figure 1). 

**Figure 2:**
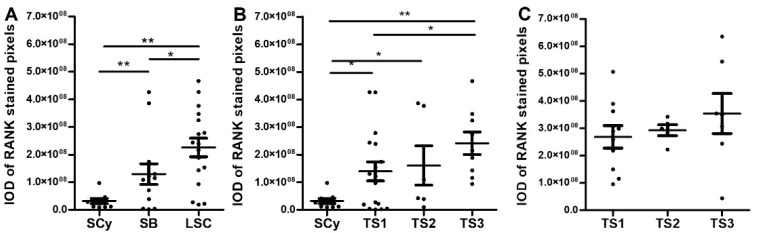
RANK protein expression in serous ovarian tumor tissues represented as IOD mean ± standard error of mean (SEM). (**A**) RANK expression values in LGSOC samples grouped attending to category. Abbreviations: IOD = integrated optical density, SCy = serous cystadenoma (*n* = 9), SB = serous borderline (*n* = 14), LSC = low-grade serous carcinoma (*n* = 32). (**B**) RANK expression values in LGSOC samples grouped by tumor stage. Abbreviations: TS1 = tumor stage 1, TS2 = tumor stage 2, TS3 = tumor stage 3. (**C**) RANK expression values in HGSOC samples grouped by tumor stage. Abbreviations: HSC = high-grade serous carcinoma (*n* = 22), TS1=tumor stage 1, TS2 = tumor stage 2, TS3 = tumor stage 3. * *p* ≤ 0.05, ** *p* ≤ 0.01 statistically significant differences between different groups after one-way ANOVA followed by Fisher LSD post-hoc test analysis.

In line with the general pattern of expression anticipated above, RANK protein staining values (integrated optical density [IOD] ± SEM, *p*-value vs. malignant condition) were significantly increased in low-grade malignant (2.17 ± 0.31 x 10^8^) when compared to borderline (1.15 ± 0.38 × 10^8^, *p* < 0.05) or benign conditions (0.37 ± 0.11 × 10^8^, *p* < 0.01) (Figure 2A). In low-grade serous EOC, RANK staining suggested a trend of gradually increased expression in parallel with tumor progression. Accordingly, when samples were stratified according to stage, the TS3 group showed significantly higher values than TS1, which in turn also had significantly higher levels than benign samples (Figure 2B). HG EOC does not present any benign or borderline conditions and represents a different serous histotype than LGSOC. It is of note to mention that HGSOC averaged more heightened expression (3.06 ± 0.27 × 10^8^) than malignant LGSOC (2.17 ± 0.31 × 10^8^) samples. When HGSOC were stratified to stage a mild trend of augmented RANK expression, an increasing stage was observed, but no significant differences were detected (Figure 2C).

#### 2.1.2. Quantitative RT-PCR Analyses

**Figure 3:**
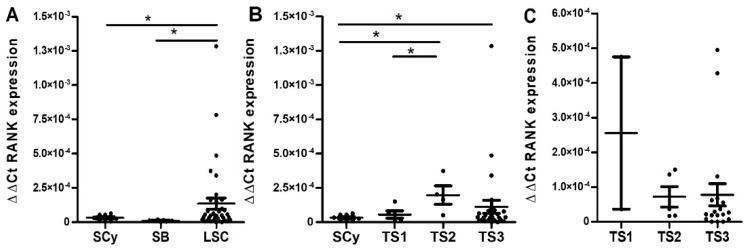
RANK mRNA expression in serous EOC represented as 2^−∆∆Ct^ RANK mean ± SEM. (**A**) RANK mRNA expression values in LGSOC samples grouped by tumor category. Abbreviations: SCy = serous cystadenoma, (*n* = 9), SB = serous borderline (*n* = 7), LSC = low-grade serous carcinoma (*n* = 30). (**B**) RANK mRNA expression values in LGSOC samples grouped by tumor stage. Abbreviations: TS1 = tumor stage 1, TS2 = tumor stage 2, TS3 = tumor stage 3. (**C**) RANK mRNA expression values in LGSOC samples grouped by tumor stage. Abbreviations: HSC = high-grade serous carcinoma (N = 26), TS1 = tumor stage 1, TS2 = tumor stage 2, TS3 = tumor stage 3. * *p* ≤ 0.05 statistically significant between-group differences after Kruskal–Wallis followed by Mann–Whitney post-hoc test.

In agreement with the plot of RANK protein expression drawn above, wt-RANK mRNA values (2^−∆∆Ct^ RANK mean ± SEM; *p*-value vs. malignant condition) were significantly higher in LGSOC malignant (1.26 ± 0.29 × 10^−4^) than in borderline (0.09 ± 0.03 × 10^−4^, *p* < 0.05) or benign (0.21 ± 0.08 x 10^−4^, *p* < 0.05) conditions (Figure 3A). When LGSOC samples were grouped according to stage, TS2 showed higher expression than TS1 and their benign counterparts. RANK values in TS3 were, however, lower than in TS2 and not significantly different than those from TS1. Therefore, in contrast to RANK protein expression, no clear trend of enhanced mRNA RANK expression associated with increased T-stage was drawn (Figure 3B). In regards to HGSOC, no trend or significant differences were found when samples were grouped according to tumor stage. In a similar fashion, no clear pattern of modulation was observed for any of the truncated RANK mRNA isoforms detected when malignant samples from either serous EOC histotypes were grouped attending to stage (Supplementary Figure S3).

### 2.2. Endometrioid EOC

#### 2.2.1. Immunohistochemical Analyses

**Figure 4:**
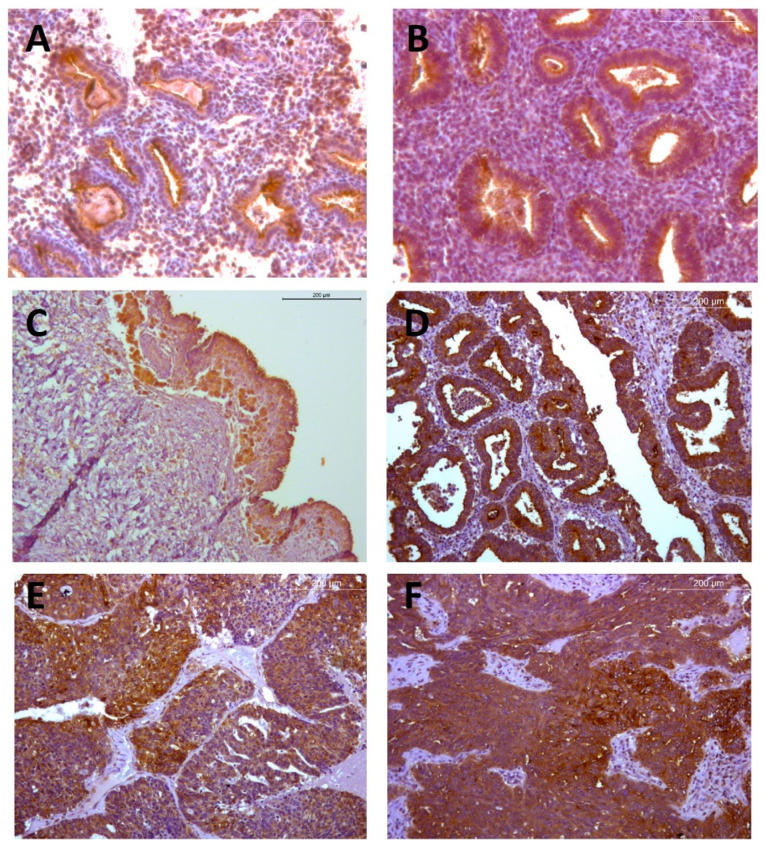
RANK staining in endometrioid ovarian tumors. Images show representative patterns of RANK immunohistochemical staining (brown) in normal endometrium, (**A**,**B**) endometrioma (benign, **C**) and endometrioid ovarian carcinoma tissue at increasing histopathological grades Grade I (**D**), Grade II (**E**) and Grade III (**F**) of malignancy. Note that cytoplasmic staining can be observed in the epithelium of benign tissue with widely extended and more intense signals in undifferentiated areas of malignant tissues. Magnifications ×100. Scale bar 200 µm.

Tissue from normal endometrial biopsies was stained as a reference to contrast RANK staining in endometrioid EOC. In eutopic endometrium, mild positive staining extended through the glandular epithelium, delineating the luminal space. In a similar fashion in benign endometrioid tissue (i.e., ectopic endometrium from endometrioma), the bulk of the signal was detected in the glandular epithelium and also extended across the stromal compartment. In endometrioid carcinoma cells, RANK staining showed intensified signal compared to benign tissue (Figure 4).

**Figure 5:**
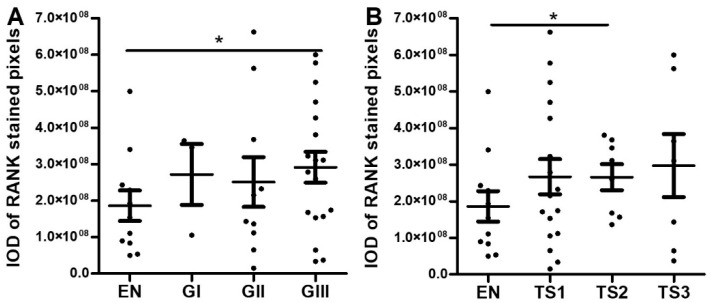
RANK protein expression in endometrioid ovarian tumor tissues represented as IOD mean ± standard error of mean (SEM). (**A**) RANK expression values in samples grouped by histological grade. Abbreviations: IOD = integrated optical density, EN= endometrioma (*n* = 11), GI = Grade I endometrioid carcinoma (*n* = 3), GII = Grade II endometrioid carcinoma (*n* = 10), GIII = Grade III endometrioid carcinoma (*n* = 18). (**B**) RANK expression values in samples grouped by tumor stage. Abbreviations: TS1 = tumor stage 1, TS2 = tumor stage 2, TS3 = tumor stage 3. * *p* ≤ 0.05, statistically significant between-group differences after one-way ANOVA followed by Fisher LSD post-hoc test analysis.

Quantitative analysis revealed almost significantly higher RANK protein staining values (IOD ± SEM, *p*-value vs. malignant condition) in malignant tumors (2.84 ± 0.63 × 10^8^) than in their benign counterparts (1.67 + 0.33 × 10^8^, *p* = 0.071). In separate comparisons, no significant difference was detected between histological grades of malignant endometrioid carcinoma samples (Figure 5A). Likewise, RANK expression did not seem to be discriminative of TS since the average RANK values were similar among stage groups (Figure 5B). Note, however, that highest grade (GIII) and stage (TS2) tumors had statistically significantly higher levels of RANK than benign samples, thus, suggesting a pattern of gradually increased RANK staining with tumor progression.

#### 2.2.2. Quantitative RT-PCR Analyses

**Figure 6:**
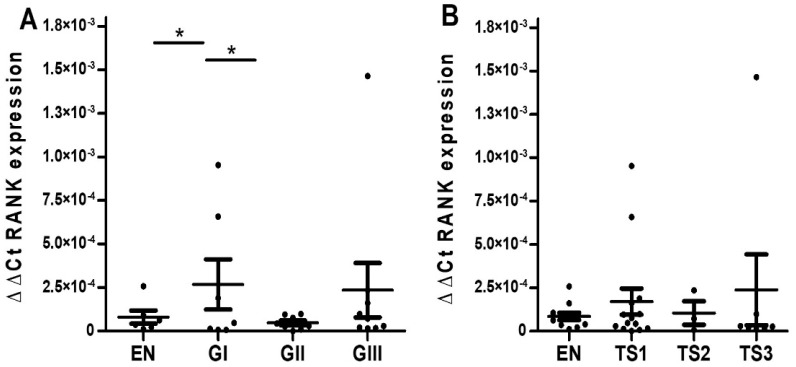
RANK mRNA expression in endometrioid ovarian tumor tissues represented as 2^−∆∆Ct^ RANK mean ± SEM. (**A**) RANK mRNA expression values in samples grouped by histological grade. Abbreviations: EN = endometrioma (*n* = 11), GI = Grade I endometrioid carcinoma (*n* = 7), GII = Grade II endometrioid carcinoma (*n* = 11), GIII = Grade III endometrioid carcinoma (*n* = 11). (**B**) RANK mRNA expression values in samples grouped by tumor stage. Abbreviations: TS1 = tumor stage 1, TS2 = tumor stage 2, TS3 = tumor stage 3. * *p* ≤ 0.05 statistically significant differences between different groups after Kruskal–Wallis followed by Mann–Whitney post-hoc test analysis.

Mirroring the pattern observed for RANK protein, wt-RANK mRNA values (2^-∆∆Ct^ RANK mean ± SEM; *p*-value vs. malignant condition) were almost significantly higher in malign samples (1.73 + 0.84 × 10^−4^) than in benign ones (0.82 + 0.32 × 10^−4^, *p* = 0.89). However, when samples were grouped according to grade or stage, we could not detect a clear increase of RANK with tumor progression. Indeed, RANK expression was significantly increased in G1, but values in the G2 group were even lower than their benign counterparts, and this rendered no clear trend for RANK expression attending to grade (Figure 6A). In a similar fashion, no clear trend was observed or significant differences between groups detected when RANK expression was plotted according to T-stage (Figure 6B). Likewise, no clear pattern of modulation was observed for any of the isoforms detected when malign samples were grouped by grade or stage (Supplementary Figure S4).

### 2.3. Mucinous EOC

#### 2.3.1. Immunohistochemical Analyses

**Figure 7:**
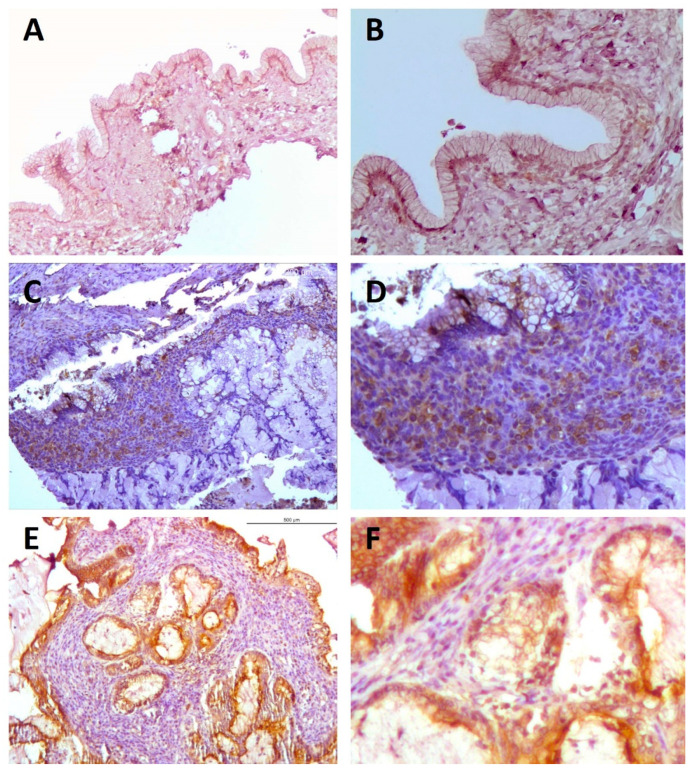
RANK staining in mucinous ovarian tumors. Images show representative patterns of RANK immunohistochemical staining (brown) in benign (mucinous cystadenoma (**A**,**B**)), borderline (mucinous borderline tumors (**C**,**D**)) and malignant (mucinous carcinoma (**E**,**F**)) mucinous ovarian tumor tissues. Images on the right side (**B**,**D**,**F**) correspond to sided magnifications of respective images (**A**,**C**,**E**) in the left column. Note that luminal epithelial cells in benign samples show cytoplasmic RANK expression mostly limited to the basal region of mucin compartment, in perinuclear areas, whereas in malignant tissue, staining is more intense and more extended over the cytoplasm. Magnifications, ×100; scale bar, 200 µm.

Overall moderate granular staining was located in stromal ovarian cells of all groups. In benign samples, a clear pattern of mild staining was detected in the luminal epithelia, mostly located in the basal cytoplasmic regions surrounding the nuclei. In borderline samples, the staining intensity increased moderately and persisted in perinuclear areas of epithelium and also appeared across the stroma. Malignant tissue presented even greater staining signaling, located in the whole cytoplasmatic compartment of epithelial cells (Figure 7).

**Figure 8:**
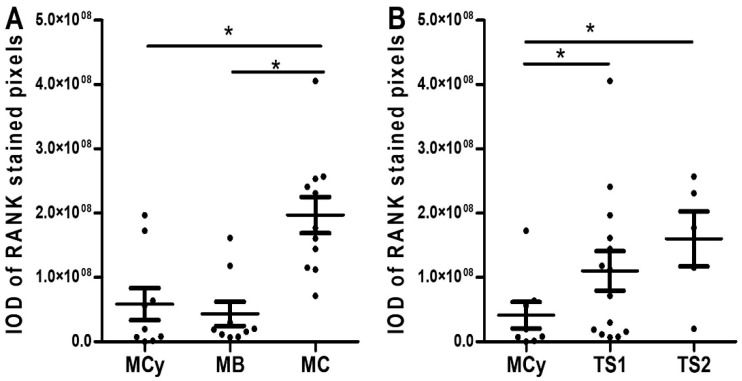
RANK protein expression in mucinous ovarian tumor tissues, represented as IOD mean ± SEM.) (**A**) RANK expression values in samples grouped by histological grade. Abbreviations: IOD = integrated optical density, MCy = mucinous cystadenoma (*n* = 9), MB = mucinous borderline tumor (*n* = 9), MC= mucinous carcinoma (*n* = 10). (**B**) RANK expression values in samples grouped by tumor stage. Abbreviations: TS1 = tumor stage 1, TS2 = tumor stage 2. * *p* ≤ 0.05 statistically significant between-group differences after one-way ANOVA followed by Fisher LSD post-hoc test analysis.

Quantification of RANK confirmed the immunohistochemical expression pattern anticipated above, with an abrupt increase in RANK staining during the transition from benign to borderline and from this to malignant conditions (Figure 8A). RANK staining also showed a progressive increase when grouped according to tumor stage (Figure 8B), suggesting a correlation with growth and spread of the main tumor.

#### 2.3.2. Quantitative RT-PCR Analyses

**Figure 9:**
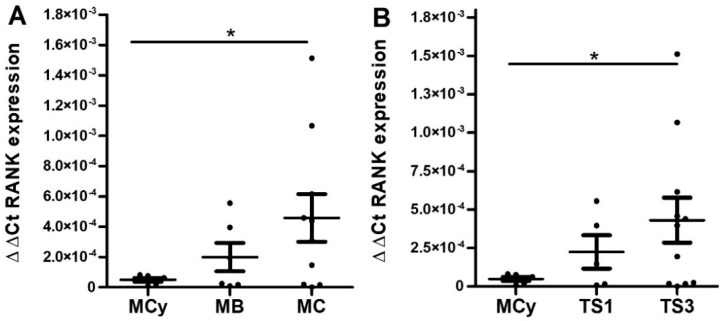
RANK mRNA expression in mucinous ovarian tumor tissues represented as 2^−∆∆Ct^ RANK mean ± SEM. (**A**) RANK mRNA expression values in samples grouped by histological grade. Abbreviations: MCy = mucinous cystadenoma (*n* = 6), MB = mucinous borderline tumor (*n* = 6), MC= mucinous carcinoma (*n* = 10). (**B**) RANK mRNA expression values in samples grouped by tumor stage. Abbreviations: TS1 = tumor stage 1, TS3 = tumor stage 3. * *p* ≤ 0.05 = statistically significant differences between different groups after Kruskal–Wallis followed by Mann–Whitney post-hoc test analysis.

Representation of RANK mRNA values according to grade (Figure 9A) and stage (Figure 9B) rendered plots mostly replicating the trend observed at the protein level. Statistically significant differences against benign conditions were detected, and a gradual increase in wt-RANK in correlation with malignization of the main tumor was drawn. In regard to truncated RANK isoforms, a notable observation is that TNFRSF11A_Δ9 was not detected in any sample. TNFRSF11A_Δ8,9 expression showed a tendency to augment with increasing stage and grade, but statistical analysis did not provide significant differences (Supplementary Figure S5).

## 3. Discussion

Our study shows that RANK expression is higher in malignant EOC than in benign or borderline tumors, a pattern consistently reproduced in a separate analysis of the three histological subtypes (serous, endometrioid and mucinous). A trend of gradual increase in RANK protein with tumor progression was suggested by the fact that the highest RANK expression was detected in the high-stage groups from serous, endometrioid and mucinous histotypes.

The pattern of RANK mRNA expression observed suggests that most of the RANK proteins detected correspond to the translation of wt-RANK, which was by far the most highly expressed isoform. In agreement with protein data, the pattern of increased RANK expression in malign vs. borderline or benign conditions was also replicated at the mRNA level. In this scenario, it seems that transcriptomic up-regulation of RANK mRNA drives the transition from benign-borderline to malignant conditions. A different issue is whether subsequent tumor progression of malignant EOCs is associated with increased RANK mRNA expression. In this regard, the pattern of gradual increase in RANK protein expression, attending to T-stage, observed in the three EOC histotypes was replicated at the mRNA level in mucinous but not in serous and endometrioid EOCs. Findings in serous and endometrioid EOCs require individual analysis in this regard. In endometrioid EOC, the association between T-stage and grade with protein RANK values, RANK was somehow weak, so a lack of correlation at the mRNA level was not totally unexpected. We have no explanation for the apparently “chaotic” expression of wt-RANK in endometrioid samples and especially in G2 samples, which showed even lower mRNA values than their benign counterparts 

In regards to serous EOC, this apparent lack of correlation between RANK mRNA and tumor progression concurs with recently published data by Wieser et al. [20], who, as in our study, found no differences in RANK mRNA expression of serous tumors grouped by stage. The apparent disagreement between RANK protein and mRNA values observed in malignant EOC of the serous histotype would be consistent with the phenotype previously observed by our group in endometrial cancer samples [16]. Copy number variations or RANK gene mutations do not seem to explain the discordance between protein and highly variable mRNA values in malignant EOT. In fact, the rate of these two phenomena is below 2%, according to NGS data of 483 serous ovarian tumors stored on TCGA. This might also be explained by the different sets of samples employed for PCR and immunohistochemical analysis. Another plausible explanation points to sustained RANK protein expression in malignant samples due to the non-transcriptional mechanism of action. In this regard, many proteins can acquire high stability while the transcriptional regulations are maintained low. Indeed, post-translational mechanisms of actions through which the half-life of proteins can be increased have been thoroughly described [21]. In addition to ubiquitination and acetylation, several other mechanisms, such as S-glycosylation, S-nitrosylation or sumoylation, have also been predicted to interfere with the degradation of protein [22]. To our knowledge, this is mostly an unexplored field in the study of RANK protein regulation.

Evaluation of the relationship between truncated RANK isoforms and cancer development/progression is an unexplored field, with the exception of seminal works by Kalafonos et al. in breast cancer [8,23]. In their studies, the authors reported that RANK-c (TNRFS11A_Δ7,8,9) attenuated breast cancer by inhibiting NF-κB activation and that its expression was inversely correlated with tumor progression. Expanding on these findings, we explored whether a similar trend could be observed in EOC and also included other RANK isoforms to characterize its expression pattern. No clear pattern or significant differences were detected for any RANK isoforms amongst malignant samples grouped by grade or stage. Truncated isoforms arise from a primary transcript (i.e., wt-RANK), which is more actively expressed in malignant than in benign/borderline conditions. Supporting this, the Pearson correlation index (data not shown) was positive for correlations between wt-RANK and either of the RANK isoforms. In this context, RANK mRNA isoform levels below the detection limit in benign conditions may merely reflect that wt-RANK expression is lower in benign vs. malignant conditions. Collectively, the results do not suggest any change or significant alteration in the expression pattern of the primary transcript or shifts in alternative splicing during tumor progression. 

Taken together, the data in ovaries follow a comparable pattern to previous findings in breast cancer. RANK expression has been associated with both lower survival and lower disease-free survival [24,25], with skeletal metastases [26], and with clinicopathological indicators, including differentiation grade [24]. This has also been reproduced in the endometrium, which, like the breast, represents another paradigmatic case of endocrine-dependent tumor [16,17,27]. This similarity between findings in tumors of breast, endometrium and now ovaries raises numerous points of interest.

The first of these regards the potential role of hormones. Extensive work in the breast has shown that the RANK/RANKL system may play a role in progestogen-induced cancer. Activation of the progesterone receptor (PR) in breast epithelium increases synthesis and release of RANKL, which then acts on RANK by a paracrine mechanism. The ensuing sequence involves activation of members of the cyclin family of proteins [28] and epithelial hyperplasia, thus increasing susceptibility to malignant transformation. Although speculative, this might also be the case of the endometrium, which is highly prone to malignant transformation as a result of hormonal stimulation. However, in this instance, the stimulator is estrogen [29], while progestogens act as protectors. If RANK/RANKL is the mechanism responsible, clarification is needed on how this conversion occurs. It is also unknown whether this occurs in the case of ovarian cancer, which has shown certain (albeit marginal) estrogen dependence [30,31,32]. 

Another important issue concerns the possible role of mutated BRCA1/2 susceptibility genes, which may represent an attractive alternative mechanism in the case of ovarian cancer. A consistent body of data has already accumulated suggesting that the oncogenic risk derived from BRCA1/2 may be conducted at least partly through the RANK/RANKL system. RANK/RANKL has been found to be involved in pre-neoplastic lesions induced by BRCA1 in the breast [33]. This hypothesis warrants further investigation, as preliminary work in ovarian cancer cell lines has shown that neither recombinant RANK ligation nor RANKL blockade with denosumab were able to limit cellular proliferation [20].

Finally, our data showing the value of RANK/RANKL as biomarkers of tumor aggressiveness are another focal point. This seems to be a constant in every area explored, whether breast, endometrium or ovary, and it is unclear whether or not the mechanisms responsible are related to those of tumorigenesis. Early work in breast cancer cell lines already confirmed that both the migratory potential of cells and the potential for bone metastases in mice were strongly influenced by RANKL, which had a promoting effect, and osteoprotegerin (OPG), which behaved as a limiting factor [34]. The association with migratory potential supports the observed relationship with metastases. In contrast, it is unclear whether the association of RANK with parameters of tumor aggressiveness, such as tumor dedifferentiation or staging, translates into the participation of RANK/RANKL/OPG in molecular mechanisms beyond those involved in the cellular migratory potential.

In summary, we found an association between clinicopathological markers of tumor aggressiveness and RANK expression levels in EOC. With small variations, the results were consistent across tumor subtypes. Our findings add to early evidence from the study of tumor series and confirm the need for further clinical research into the role of RANK/RANKL in ovarian cancer.

## 4. Materials and Methods

### 4.1. Sample Description

To investigate the prognostic value of RANK protein expression, we purchased a tissue microarray (TMA) commercially available from Abcam (catalog number ab178250, Cambridge, UK) as the main source of benign, borderline and malignant EOC. The TMA contained 228 independent 1.1 mm width paraffin-embedded tissue spots representative of different ovarian pathologies. Of the 228 spots, 8 corresponded to normal ovarian sections and 154 corresponded to EOC of the three different subtypes; and of these latter, 114 contained complete TNM-stage (TS) and tumor grade data. TMA was completed with 20 samples from the Pathology Department at our center and corresponded to benign forms of serous (*n* = 5) and endometrioid (*n* = 11) tumors, as well as malignant forms of mucinous carcinoma (*n* = 4). Thus, a total of 135 cases were finally included in the study and classified by histological subtype, as detailed in Table 1. In addition, normal fallopian tubes (*n* = 8) and endometrial samples (*n* = 8) from our archive were also sectioned and processed for IHC analysis.

To investigate whether the amount of wt-RANK mRNA and truncated isoforms was related to prognostic factors of ovarian cancer, four commercially available (catalog number HORT301, HORT302, HORT303, HORT 304) TissueScanTM cDNA ovarian cancer array (TcDA) plates were purchased from OriGene (Rockville, MD, USA). Each plate contained five identical sets of 48 tissues covering normal (ovarian tissue) and disease stages representative of four different ovarian pathologies. Out of the 196 different well plate cDNAs, 129 corresponding to EOCs of the three different subtypes contained complete TNM-stage (TS) and tumor grade data. Added to the original TcDA PCR plates were exogenous cDNAs (2 µL, containing 100 ng of cDNA equivalent pipetted per well) from 36 cases stored at the Pathology Department at our center. These corresponded to benign forms of serous (*n* = 7) endometrioid (*n* = 11) and mucinous tumors (*n* = 6), as well as borderline (*n* = 6) and malign (*n* = 4) forms of the latter. Therefore, a total of 168 cases were finally included in the study and classified by histological subtype, as detailed in Table 2. 

The Human Research Ethics Review Board of INCLIVA approved the entire protocol (project code 2020/124). Benign, borderline and malignant EOCs were classified based on WHO criteria [35] as low-grade serous ovarian carcinoma (LGSOC), high-grade serous ovarian carcinomas (HGSOC), endometrioid carcinomas and mucinous carcinoma. Following the same WHO criteria, borderline and malignant cases of endometrioid, mucinous and LGSOC, as well as malignant forms of HGSOCs, were categorized according to their TS as TS1, TS2, TS3 and TS4 for comparative purposes. Furthermore, endometrioid carcinomas were classified attending to histopathological grade (Grade I, II or III) LGSOC, HGSOC and mucinous malignant carcinomas do not present any histopathological grade subclassification [35]. Normal fallopian tubes and endometrial biopsies were also included as controls for IHC purposes.

### 4.2. Immunohistochemical Quantification of RANK Protein 

#### 4.2.1. Immunohistochemical Detection 

RANK was identified by incubating 4 µm sliced sections with RANK/TNFRSF11A antibody (MAB6831—R&D Systems Inc., Minneapolis, MN, USA) at 1:400 in Dako Antibody Diluent overnight at 4 °C. The next day, colorimetric staining was revealed using Dako REAL© EnVision© Peroxidase/DAB+, Rb/Mo Detection System (catalog number K500711, Glostrup, Denmark), as previously optimized by Gomez et al. [16]. 

#### 4.2.2. Image Acquisition

Samples were photographed at 10X and 20X using a Nikon Eclipse E400 microscope (Nikon, Tokyo, Japan) connected to Leica Application Suite Version 4.9.0 (Leica Microsystems Ltd. Software, Heerbrugg, Switzerland). For quantitative analysis purposes, a total of four random images per sample were acquired in the area of interest, as previously reported by our group [16,18]. 

#### 4.2.3. RANK IHC Staining Signal Analysis

Image Pro Plus 6.0 software (Media Cybernetics Inc., Silver Spring, MD, USA) was used to quantify the stained RANK area following the methodology previously published by our group [16,18]. In brief, the area of interest covered by tissue was delineated, and the intensity of stained pixels was quantified using the Optical Density (OD) tool of the software. OD values were inverted to create a scale directly proportional to staining intensity. The sum of inverted OD values of the stained pixel, also known as integrated optical density (IOD), was used to define the intensity of RANK staining in each area of interest (i.e., per image). The IOD average of four areas of interest was used to define RANK intensity in each sample section. 

### 4.3. QF-RT-PCR Quantification of Wildtype and Truncated RANK mRNA Isoforms

OriGene ovarian cancer array supplemented plates were loaded into each well with 10 µL of a master mix stock solution containing 2× SYBR green mix (Life Technologies, Carlsbad, CA, USA), water and specific primers at 0.05 µm final concentration. The sequence of primers for β-actin, wt-RANK (TNFRSF11A) and its variant truncated RANK isoforms have been previously described [8]. Real-time PCR was performed using an ABI PRISM 7500 Sequence Detection System (Perkin Elmer Corp., Norwalk, CT, USA) according to the manufacturer’s instructions, with a heated lid (105 °C), an initial denaturation step at 95 °C for 10 min, followed by 40 cycles of 95 °C for 15 s and 60 °C for 1 min. Relative expression level of each RANK isoform was calculated with the comparative 2∆∆Ct method, where ∆Ct = Ct(target) − Ct(control), ∆∆Ct = Ct(target) − Ct(calibrator) and all samples were normalized to the β-actin gene. 

### 4.4. Statistics and Figure Representation

Statistical analysis and graph representation were performed using SigmaPlot 14.0 (Systat Software, San Jose, CA, USA) and GraphPad Prism (GraphPad Software, San Diego, CA, USA) software. Data were expressed as mean ± standard error of the mean (SEM). For multiple comparisons, cases were grouped according to histological grade and tumor stage, and one-way ANOVA with post-hoc Fisher LSD or non-parametric Kruskal–Wallis followed by post-hoc Mann–Whitney tests were employed to detect specific between-group differences. Statistical significance was defined as *p* ≤ 0.05. 

## Figures and Tables

**Table 1:** Sample size, histological classification and categories of epithelial ovarian cancers intended for immunohistochemical analysis. The table shows classification of epithelial ovarian cancers included in the study by histotype: (serous (low-grade, high-grade), endometrioid and mucinous), category (benign, borderline and malignant) and T-stage (TS1, TS2, TS3). Note that as an unusual diagnosis, endometrioid borderline cases were not available, and were thus excluded from the study. High-grade EOCs do not present benign or borderline categories. Abbreviations are indicated in brackets.

	Serous						Endometrioid			Mucinous		
	Low			High								
**B** **enign**	**9**						11			9		
**Borderline**	**TS1**	**TS2**	**TS3**				0			**TS1**	**TS2**	**TS3**
	10	3	1							8	1	0
**Malignant**	**TS1**	**TS2**	**TS3**	**TS1**	**TS2**	**TS3**	**TS1**	**TS2**	**TS3**	**TS1**	**TS2**	**TS3**
	8	3	8	10	5	7	17	8	7	6	4	0

**Table 2:** Sample size, histological classification and categories of epithelial ovarian tumors intended for PCR analysis. The table shows classification of epithelial ovarian cancers (EOC) included in the study by histotype (serous (low-grade and high-grade), endometrioid and mucinous) category (benign, borderline and malignant) and T-stage (TS1, TS2, TS3). Note that as an unusual diagnosis, endometrioid borderline cases were not available, and were thus excluded from the study. High-grade EOCs do not present benign or borderline categories. Abbreviations are indicated in brackets.

	Serous						Endometrioid			Mucinous		
	Low			High								
**B** **enign**	**9**						11			6		
**Borderline**	**TS1**	**TS2**	**TS3**				0			**TS1**	**TS2**	**TS3**
	3	2	2							2	0	4
**Malignant**	**TS1**	**TS2**	**TS3**	**TS1**	**TS2**	**TS3**	**TS1**	**TS2**	**TS3**	**TS1**	**TS2**	**TS3**
	2	2	26	2	5	19	14	3	7	3	0	7

## Data Availability

The data presented in this study are available on reasonable request from the corresponding author.

## Supplementary Materials

The following are available online at https://www.mdpi.com/article/10.3390/ijms23031742/s1.
