# Supplementary material for: Histological Grade and Tumor Stage Are Correlated with Expression of Receptor Activator of Nuclear Factor Kappa b (Rank) in Epithelial Ovarian Cancers
Source: Int J Mol Sci. 2022 Feb 3;23(3):1742. doi: 10.3390/ijms23031742 (PMC8836022; doi:10.3390/ijms23031742)
Supplement: Supplementary file 1 [file ijms-23-01742-s001.zip › ijms-1463168-supplementary.pdf]

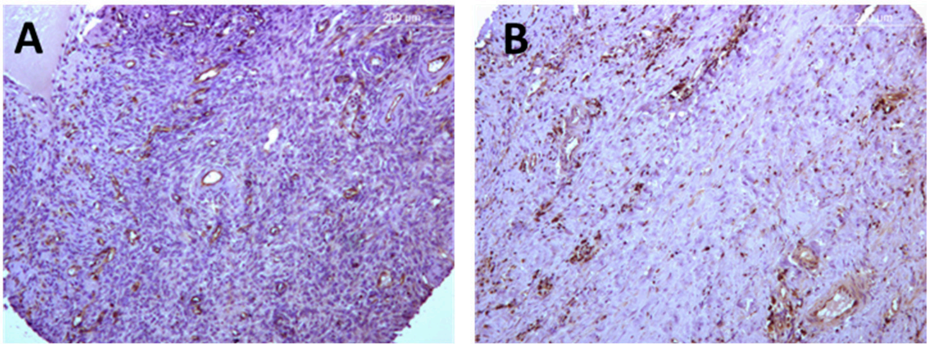

Figure S1. RANK staining in control ovaries.

Pictures (A, B) correspond to **tissue** spots of the TMA containing representative normal ovarian sections immunostained against RANK (brown color). Note mild staining mostly restricted to vessels and scarce stromal cells. Magnifications x10= Scale bar 200µm.

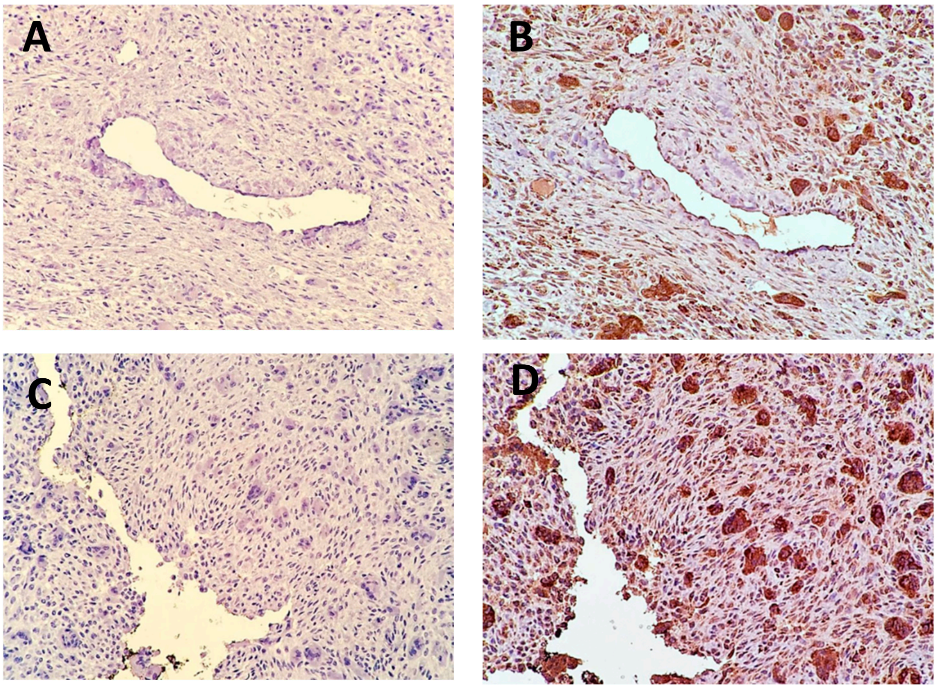

Figure S2. RANK staining in positive tissue controls

Pictures correspond to RANK immunohistochemical staining (brown) of giant cells of bone tumor tissues (column on the right). Siding images on the left side (A, C) correspond to serial sections (negative controls) in which the primary RANK antibody was omitted during IHC. Note clear intense staining in Giant Cells and mild signal in stromal cells of tissue (right panel, B and D).

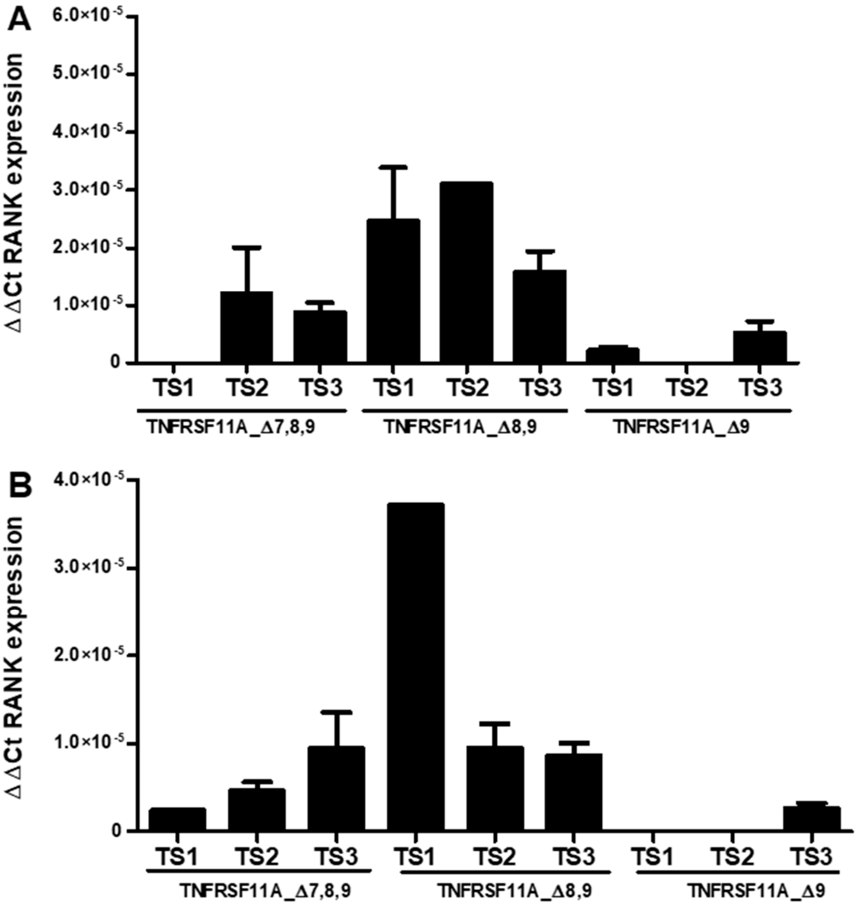

Figure S3. Expression of truncated RANK mRNA isoforms (TNFRSF11A_Δ7,8,9, TNFRSF11A_Δ8,9 and TNFRSF11A_Δ7,8,9) in serous EOC tissues, represented as 2-ΔΔCt RANK mean + SEM. (A) Truncated RANK mRNA isoform expression values in low grade serous carcinoma (A) and high grade serous carcinoma (B) grouped by tumor stage. Abbreviations: TS1=Tumor Stage 1, TS2=Tumor Stage 2, TS3=Tumor Stage 3. No statistically significant differences between-group were detected after Kruskal–Wallis analysis.

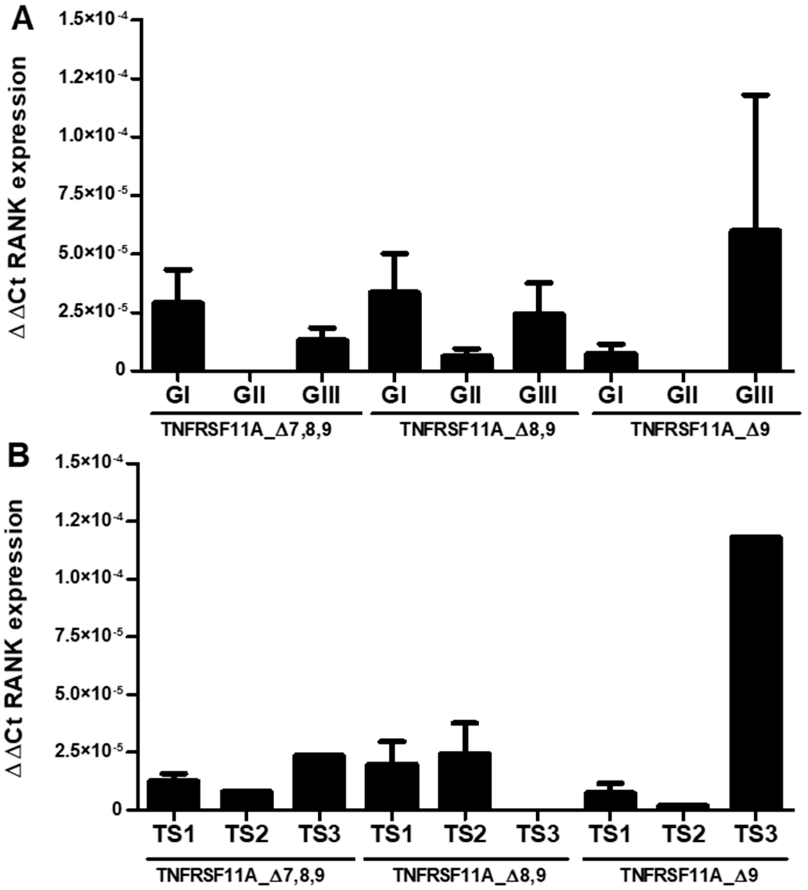

Figure S4. Expression of truncated RANK mRNA isoforms (TNFRSF11A_Δ7,8,9, TNFRSF11A_Δ8,9 and TNFRSF11A_Δ7,8,9) in endometrioid EOC tissues, represented as 2-ΔΔCt RANK mean + SEM. (A) Truncated RANK mRNA expression values in samples grouped by histological grade. Abbreviations: GI= Grade I endometrioid carcinoma, GII= Grade II endometrioid carcinoma and GIII= Grade III endometrioid carcinoma. (B) Truncated RANK mRNA isoform expression values in samples grouped by tumor stage. Abbreviations: TS1=Tumor Stage 1, TS2=Tumor Stage 2, TS3=Tumor Stage 3. No statistically significant between-group differences were detected after Kruskal–Wallis analysis.

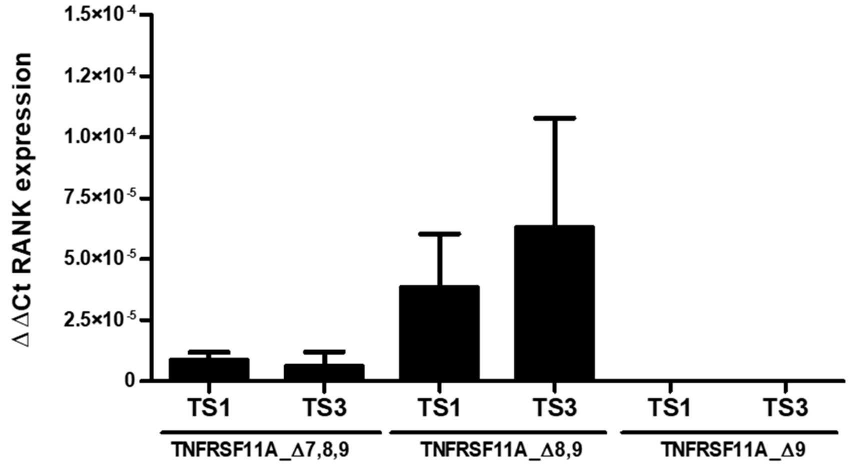

Figure S5. Expression values of truncated RANK mRNA isoform (TNFRSF11A_Δ7,8,9, TNFRSF11A_Δ8,9 and TNFRSF11A_Δ7,8,9) in samples grouped by tumor stage in mucinous EOC tissues represented as 2-ΔΔCt RANK mean + SEM. Abbreviations: TS1=Tumor Stage 1, TS3=Tumor Stage 3. Truncated RANK mRNA isoform. No statistically significant between-group differences were detected after Kruskal–Wallis analysis.
